# ﻿*Ferulagroessingii* (Apiaceae), a new synonym of *Ferulalicentiana* var. *tunshanica*

**DOI:** 10.3897/phytokeys.254.145845

**Published:** 2025-03-18

**Authors:** Lei Yang, Wen-Jun Li

**Affiliations:** 1 State Key Laboratory of Ecological Safety and Sustainable Development in Arid Lands, Xinjiang Institute of Ecology and Geography, Chinese Academy of Sciences, Urumqi 830011, China Chinese Academy of Sciences Urumqi China; 2 Xinjiang Key Lab of Conservation and Utilization of Plant Gene Resources, Urumqi, 830011, China Xinjiang Key Lab of Conservation and Utilization of Plant Gene Resources Urumqi China; 3 College of Resources and Environment, University of Chinese Academy of Sciences, Beijing, 100049, China University of Chinese Academy of Sciences Beijing China

**Keywords:** Apiaceae, China, *Ferula*, new synonym, taxonomy

## Abstract

A comprehensive evaluation of the diagnostic characters employed in distinguishing *Ferulagroessingii* from F.licentianavar.tunshanica has led to the conclusion that the two taxa are indeed conspecific. As a result, *F.groessingii* is hereby recognized as a new synonym of F.licentianavar.tunshanica. This reclassification is supported by a comprehensive comparison of taxonomic features, morphological evidence, and distribution data. The study confirms that key morphological traits including plant height, a hairy coat of stems and leaves, number of inflorescences, and fruit vittae, are critical for the identification of this species complex.

## ﻿Introduction

*Ferula* L. is one of the largest genera in the Apiaceae family, encompassing approximately 220 species globally (https://powo.science.kew.org/results?q=ferula). It is mainly distributed in the southern part of Europe, Northern Africa, Central Asia, and the Mediterranean region, with Central Asia being the biodiversity hotspot of this genus (Pimenov and Leonov 1993).

**Figure 1.**
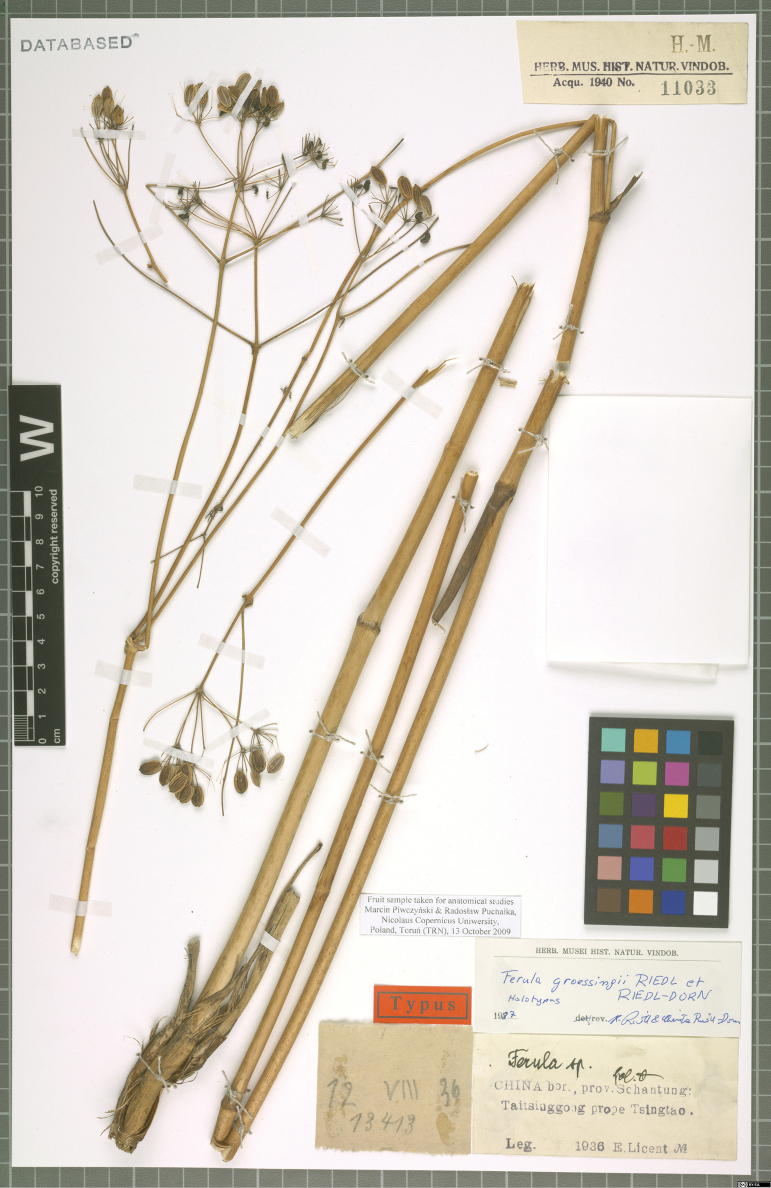
The holotype specimen of *F.groessingii* (https://www.jacq.org/detail.php?ID=191435).

*Ferulagroessingii* Riedl & Riedl-Dorn was originally described as a new species endemic to China based on a single fruiting collection made by Licent from Taiqinggong in Qingdao, Shangong Province (Fig. 1). In the protologue, Riedl and Riedl-Dorn (1987) compared *F.groessingii* with *F.bungeana*Kitagawa (1956) and *F.rigidula*Candolle (1830), but they overlooked *F.licentiana*Handel-Mazzetti (1933) and *F.tunshanica* Anon. (Jiangsu Institute of Botany 1982), which are distributed in Shanxi, Shaanxi, Henan, Hebei, Hubei, Shandong, Anhui and Jiangsu provinces of China (Fig. 3). Furthermore, since its publication, the name has not received attention and was not included in the Flora Reipublicae Popularis Sinicae (Shen 1992) or in the Flora of China (She and Watson 2005). The name *F.groessingii* reappeared only in Pimenov’s checklist of the Apiaceae in China (Pimenov 2017). However, for the 30 years following its publication, no other literature mentioned this species name.

During our studies and exploration of the specimens of *Ferula* in China, we found that *F.groessingii* is morphologically very similar to F.licentianavar.tunshanica. The objective of this study is to confirm the relationship between *F.groessingii* and F.licentianavar.tunshanica.

## ﻿Material and methods

We collected specimens of *Ferulagroessingii* from the type locality in 2023 to 2024. These specimens were deposited at the Herbarium of Xinjiang Institute of Ecology and Geography, Chinese Academy of Sciences (XJBI). For the detailed collection information, including coordinates, please refer to the “Specimens examined” section. We carefully checked and studied the specimens from NAS and PE in person, while materials from KUN and WUK were examined through photographs on the Chinese Virtual Herbarium (CVH) website (https://www.cvh.ac.cn/index.php). We also conducted detailed comparisons of F.licentianavar.tunshanica plant images available on the Plant Photo Bank of China (PPBC) website (https://ppbc.iplant.cn/sp/24536). Photographs were taken in the field using a Nikon Z7 II camera. Hairs in the stems and leaves were examined using a dissecting microscope Phenix XTL-165 in the laboratory. In order to prepare tissue sections, the mature fruits were softened before being embedded in paraffin. Following staining, the sections were mounted with neutral balsam, observed under a microscope, and photographed for analysis. Additionally, we compared the protologue of *F.groessingii* with our observed results.

## ﻿Results

**Figure 2.**
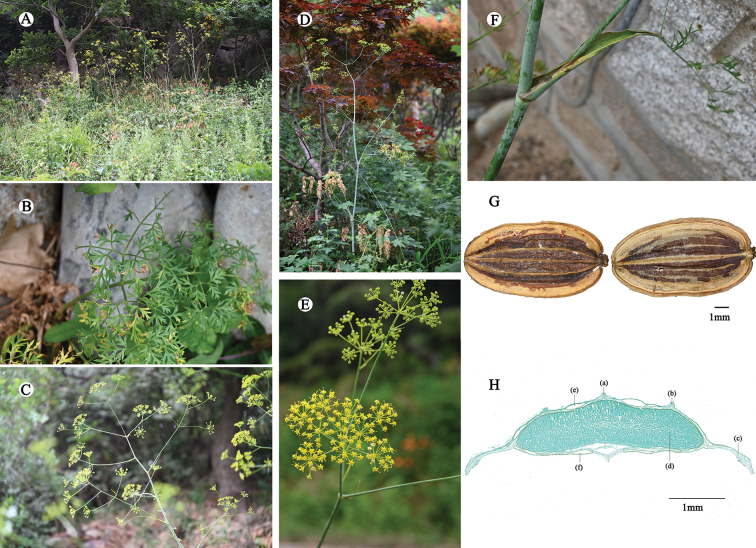
Morphology of *F.groessingii***A** habitat **B** basal leaves **C** inflorescence **D** individual **E** flowering stage **F** cauline leaves **G** mericarps, dorsal and commissural aspects **H** cross section of mericarp: (a) median rib, (b) lateral rib, (c) marginal rib, (d) endosperm, (e) vallecular vitta, (f) commissure vitta. Scale bar: 1 mm.

**Figure 3.**
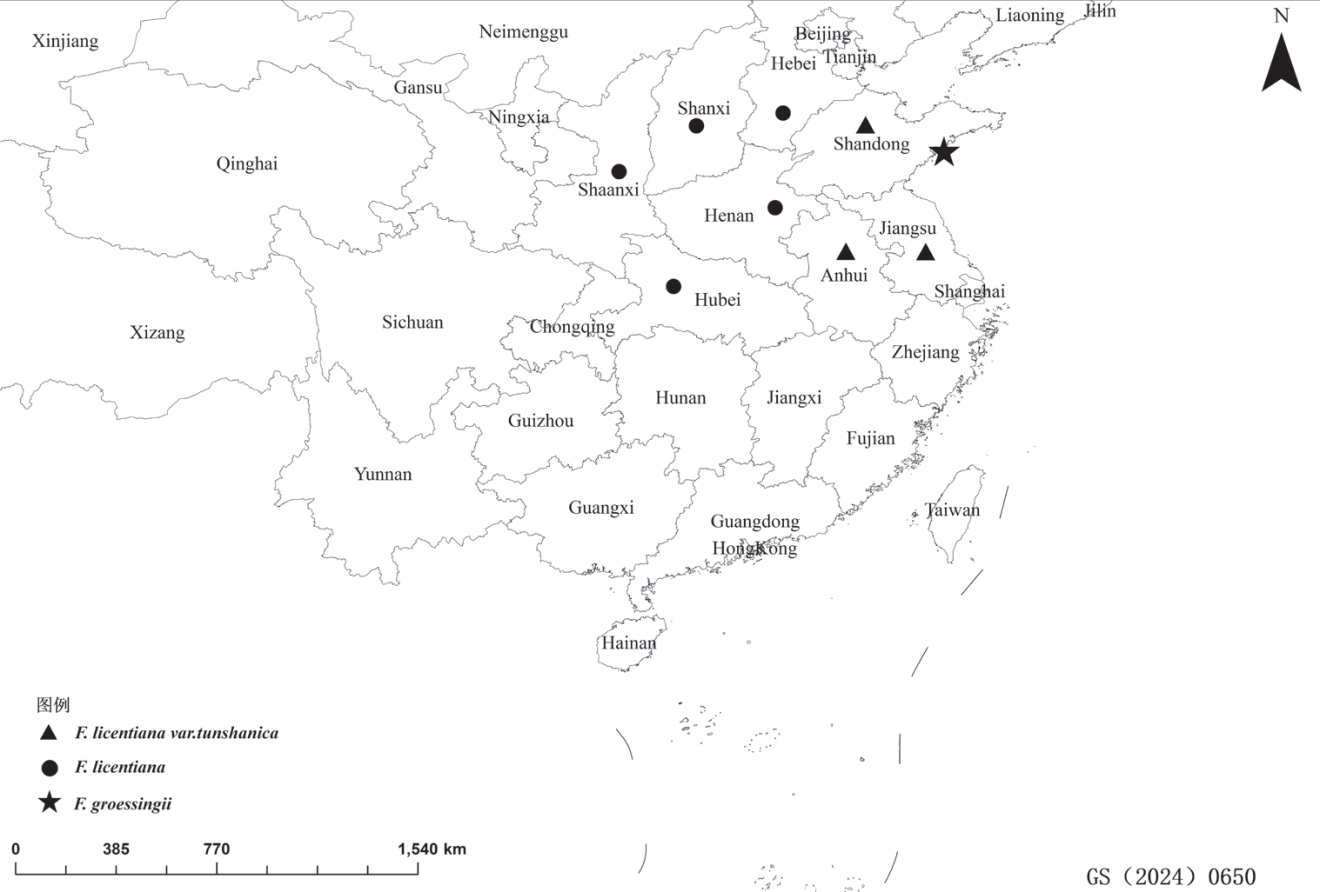
Distribution of *F.licentiana*, F.licentianavar.tunshanica and *F.groessingii*. The map is based on a standard map downloaded from the National Natural Resources Ministry’s Standard Map Service website. The boundaries of the base map have not been modified.

We found a clear resemblance between *F.groessingii* and F.licentianavar.tunshanica (Figs 1, 2, Table 1). Field sampling conducted at the type locality of *F.groessingii* and its detailed comparisons with F.licentianavar.tunshanica revealed that its characters are nearly identical. Key diagnostic morphological features such as the plant being glabrous throughout, with an average height of about 2 m, a solitary and slender stem, as well as the morphology of the inflorescence and fruit vittae were found to be consistent between two taxa (She and Watson 2005). The distribution of *F.groessingii* matches completely with that of F.licentianavar.tunshanica. The latter is predominantly found on sunny mountain slopes in Shandong, Anhui and Jiangsu provinces (Fig. 3). Consequently, based on these findings, we propose *F.groessingii* be treated as a synonym of F.licentianavar.tunshanica.

**Table 1.** Morphological comparisons of *F.licentiana*, F.licentianavar.tunshanica and *F.groessingii*.

Characters	F.licentianavar.licentiana	F.licentianavar.tunshanica	*F.groessingii*
**Habit**	1.2–1.8 m	Smaller	1.5–1.7 m
**Stem**	stem solitary, slender, usually flexuose, paniculate-branched, lower branches alternate, upper branches verticillate		stem glabrous, with a diameter of only 10 mm near the base, branched from the upper part
**Leaves**	both surfaces glabrous, upper leaves reduced, bladeless, sheaths lanceolate, embracing		both surfaces of the leaves are smooth and glabrous
**Inflorescences**	terminal umbel short-pedunculate, lateral umbels 1–3, simple or opposite, exceeding terminal, rays 7–11, umbellules 7–11flowered.	rays fewer, 3–7	short peduncle, with 5–7 rays; lateral umbels are often opposite, with long peduncles that exceed the central umbel, and have 7–9 rays.
**Fruit**	oblong or oblong-obovate, 10–15 mm; lateral broadly winged	fruits are also smaller, less than 10 mm	elliptical or oblong, measuring 4.5 × 8 millimeters to 5.5 × 9 millimeters, with a thickness of 0.5–0.7 millimeters. The top of the fruit is slightly notched, with a filiform dorsal rib, and the vittae in each furrow are very broad.
**Vittae**	3–4 per furrow, 4–8 toward commissure	1–3 per furrow, 4–6 toward commissure	commissural vittae are 4–6

## ﻿Discussion

The scientific name F.licentianavar.tunshanica has an intriguing taxonomic backstory. It was initially published as *F.tunshanica* Su in the Flora of Jiangsu (Vol. 2: 935. 1982), where “Su” was explicitly stated to represent a collective author (Jiangsu Institute of Botany). Subsequent treatments, whether the invalid reduction to a variety by Liu et al. (1987) or the inadvertent valid publication (Shen 1992; Yu et al. 2010), correctly cited the name authorship. However, in the Flora of China (She and Watson 2005), “Su” was mistakenly interpreted as referring to Song-Wang Su (S.W. Su) who confirmed not to be the author of this taxon, a misinterpretation that has persisted to this day. With no traceable natural person attributable to the name and in accordance with ICN 2018 Art. 46.9 Ex. 45, the correct citation for *F.tunshanica* should be *Ferulatunshanica* Anon. (in Fl. Jiangsu 2: 935. 1982).

### ﻿Taxonomic treatment

#### ﻿ Ferula licentiana var. tunshanica

Taxon classificationPlantaeApialesApiaceae

(Anon.) R.H.Shan & Q.X.Liu ex K.M.Shen, Fl. Reipubl. Popularis Sin. 55(3): 114 (1992).

EF8DE233-CC83-5E75-9FD2-CDDEEF0AE8AE

≡ Ferulatunshanica Anon. in Fl. Jiangsu 2: 935 (1982).
= Ferulagroessingii Riedl & Riedl-Dorn, Linzer Biol. Beitr. 19(2): 485 (1987), syn. nov. Type. CHINA. Shandong province: Qingdao city, Taiqinggong, 12 August 1936, Licent E 13413 (holotype W, image!)

##### Type.

China. • Jiangsu province: Tongshan, Maocun, 22 June 1974, Wen-Zhe Fang & Ping-Ping Ling et al. 74020 (holotype: NAS00042894!).

##### Specimens examined.

**China.** • **Shandong**: city. Qingdao, county Laoshan (36°08.17'N, 120°40.85'E), 18 Jun 2023, Wen-jun Li & Lei Yang LS20230618001 (XJBI, barcode XJBI00162552!; XJBI00162553!; XJBI00162554!; XJBI00162555!; XJBI00162556!; XJBI00162557!); city. Jinan, Hushan Forest Park, 11 Jun 2015, Xiao-wei Xin Lilan794 (KUN, image! barcode KUN1480754); city. Jinan, Foyu Valley, 8 Jun 1977, Chang-qi Yuan 83 (NAS, barcode NAS00021792!; NAS00021793!; NAS00021795!; NAS00021796!; NAS00021801!); city. Jinan, 12 Jun 1964, s.n. 64036 (NAS, barcode NAS00021719!; NAS00021720!). • **Henan**: city. Lingbao, Jiaoyuan Valley, 27 Jun 1974, Zhi-xin Hu 17213 (WUK, image! barcode WUK0416132; WUK0416133; WUK0297545). • **Hubei**: city. Zaoyang, county Shangdang, mountain Yazishan, 13 Jun 2018, Shen-lan Li GanQL1240 (KUN, image! barcode KUN1457994). • **Jiangsu**: mountain Tongshan, 10 Jul 1976, Ren-hua Shan 7607E (NAS, barcode NAS00021800!). • **Shannxi**: mountain Huashan, Yuquanyuan, 26 May 1956, Kun-jun Fu & Ben-zhao Guo 10101 (WUK, image! barcode WUK0092684); county Huayin, mountain Huashan, 24 Jun 1974, Kun-jun Fu 17207 (WUK, image! barcode WUK0416139; WUK0416140; WUK0297551); mountain Huashan, Suoluopin, 10 May 1961, Zhiwuxi 104 (WUK, image! barcode WUK0483271); mountain Huashan, Yukou, 1 Jun 1956, Kun-jun Fu & Ben-zhao Guo 10148 (WUK, image! barcode WUK0099592); county Huayin, Xianyu, 23 Jun 1974, Kun-jun Fu 17205 (WUK, image! barcode WUK0416128; WUK0416129; WUK0297553); county Huayin, Huayingongshe, 28 Jun 1974, Kun-jun Fu 17215 (WUK image!, barcode WUK0416130; WUK0416131). • **Shanxi**: county Huairen, 24 May 1938, W.Y.Hsia 4373 (PE, barcode PE00756177!).

## Supplementary Material

XML Treatment for Ferula licentiana var. tunshanica

## ﻿Additional information

### Author ORCIDs

Lei Yang https://orcid.org/0009-0003-8888-5893

Wen-Jun Li https://orcid.org/0000-0002-2932-0783

### Data availability

All of the data that support the findings of this study are available in the main text.
